# High Folic Acid Intake during Pregnancy Lowers Body Weight and Reduces Femoral Area and Strength in Female Rat Offspring

**DOI:** 10.1155/2013/154109

**Published:** 2013-05-27

**Authors:** Pedro S. P. Huot, David W. Dodington, Rebecca C. Mollard, Sandra A. Reza-López, Diana Sánchez-Hernández, Clara E. Cho, Justin Kuk, Wendy E. Ward, G. Harvey Anderson

**Affiliations:** ^1^Department of Nutritional Sciences, Faculty of Medicine, University of Toronto, Toronto, Canada M5S 3E2; ^2^Centre for Bone and Muscle Health, Faculty of Applied Health Sciences, Brock University, St. Catharines, Canada L2S 3A1

## Abstract

Rats fed gestational diets high in multivitamin or folate produce offspring of altered phenotypes. We hypothesized that female rat offspring born to dams fed a gestational diet high in folic acid (HFol) have compromised bone health and that feeding the offspring the same HFol diet attenuates these effects. Pregnant rats were fed diets with either recommended folic acid (RFol) or 10-fold higher folic acid (HFol) amounts. Female offspring were weaned to either the RFol or HFol diet for 17 weeks. HFol maternal diet resulted in lower offspring body weights (6%, *P* = 0.03) and, after adjusting for body weight and femoral length, smaller femoral area (2%, *P* = 0.03), compared to control diet. After adjustments, HFol pup diet resulted in lower mineral content (7%, *P* = 0.01) and density (4%, *P* = 0.002) of lumbar vertebra 4 without differences in strength. An interaction between folate content of the dam and pup diets revealed that a mismatch resulted in lower femoral peak load strength (*P* = 0.01) and stiffness (*P* = 0.002). However, the match in folate content failed to prevent lower weight gain. In conclusion, HFol diets fed to rat dams and their offspring affect area and strength of femurs and mineral quantity but not strength of lumbar vertebrae in the offspring.

## 1. Introduction

Osteoporosis is a major public health concern in North America and affects as many as 2 million Canadians [1] and 40 million Americans [2]. The financial burden of long-term, hospital, and chronic care of osteoporosis is estimated to be $2.3 billion dollars per year in Canada [3] and greater than $15 billion dollars per year in the United States [4]. Adult bone health and risk of osteoporosis is dictated, in part, by whether individuals achieve peak bone mass by young adulthood [2, 5]. Peak bone mass is controlled by genetics as well as lifestyle factors including diet and physical activity. Epidemiological evidence suggests that many children from families with history of fractures have lower bone mass, and therefore higher risk for fractures [2]. Moreover, appropriate nutrition during pregnancy and in early childhood is essential in maintaining bone health and can alter the trajectory of achieving peak bone mass (as reviewed in [5–7]). 

The interest in folic acid intake during pregnancy and childhood and its effects on bone health is twofold. First, higher intake of folic acid is associated with higher bone mineral density (BMD) of postmenopausal women older than 50 [8, 9] and is associated with a lower risk of fractures in men and women older than 65 [10, 11]. Second, early diet may modulate the risk of developing diseases in adulthood [12–14]. This phenomenon is often referred to as nutritional programming. Recent data from both animal and human studies have led to the concern that excess multivitamins or folic acid alone during pregnancy may increase the risks of developing chronic diseases, including cancer growth [15] and the metabolic syndrome [12, 13]. Folic acid supplementation during pregnancy and lactation at 2.5-fold higher levels than control diet results in female rat offspring with lower body weights at weaning and at 50 days of age [16]. However, there is inconsistency in the relationship between maternal folate intake and birth weights. A study of pregnant women in Mexico observed a positive correlation between folic acid intake and infant birth weight [17]. In contrast, during the second trimester of pregnancy there was no association between maternal folate status and birth weight, or maternal folate intake levels (which included the use of supplements) and birth weight, in well-nourished Norwegian women [18]. Although not yet examined in humans, diets rich in methyl groups [19] or diets high in multivitamins fed to rats during pregnancy markedly increase body fat [12], alter metabolic phenotype of the offspring [12–14, 20–22], and disrupt expression of hypothalamic genes involved in food intake regulation [14, 22]. This suggests that programming by micronutrients can impact many different areas of health and that the nutritional status of the mother may be a factor.

New studies have emerged focusing on the role of folic acid in bone development *in utero* and in childhood, indicating a possible beneficial role. Higher folate status in pregnant women was positively associated with higher total and spinal BMD in children at 6 years old [23] and higher BMD and bone mineral content (BMC) in the spine subregion in children at 9 years old [24]. Furthermore, length of *in utero *exposure to folic acid is important. Women who consumed 1 mg of folic acid daily (2.5-fold the required levels) for their entire pregnancy had newborns with better bone health attributes compared to newborns from women that consumed the supplement only during the first and second trimesters [25]. Lastly, pregnant women are recommended to take folic acid supplements containing 2.75-fold to 12.5-fold [26–28] the estimated requirements to prevent neural tube defects, congenital disorders, and adverse pregnancy outcomes [29–31]. However, the long-term consequences of these higher levels of intake are poorly understood.

The objective of this study was to determine the effect of a high folic acid (HFol; 10-fold) maternal diet on body weight gain and bone health in female offspring. Further, we fed offspring an HFol diet in order to test the Predictive Adaptive Response hypothesis (PAR) [32]. The PAR states that the developing individual is constantly responding to intrauterine stimuli and, correspondingly, will make adaptations to its physiology to predict its postnatal environment. If this prediction is incorrect because the postnatal and gestational environments do not match, then the risk of developing adverse effects in later life increases. Conversely, matching the postnatal and gestational environments will decrease the risk of developing adverse effects. 

We hypothesized that female offspring born to dams fed a HFol diet during pregnancy have lower BMC, BMD, and bone strength, independent of lower body weights. We further hypothesized that matching the folic acid content of the weaning diet with the maternal diet would attenuate the adverse effects of the HFol maternal diet. 

## 2. Methods

### 2.1. Animals and Diet

First time pregnant (2nd-3rd day of pregnancy) Wistar rats were obtained from Charles River Laboratories (Montreal, QC, Canada). Two groups of rats (*n* = 10/group) were randomly assigned to the AIN-93G control diet with the recommended amount of folic acid (RFol, 2 mg folic acid/kg diet) [33] or a modified AIN-93G diet supplemented with 10-fold the recommended amount of folic acid (HFol, 20 mg folic acid/kg diet) during pregnancy. The dose of 10-fold is within the range of folic acid supplementation that pregnant women are prescribed. We have previously reported that supplementation of folic acid and other multivitamins up to 10-fold is not toxic to either the dams or the offspring [12, 13, 20].

The RFol diet contained 2 mg folic acid/kg of diet (Table 1). Therefore, we added 18 mg of folic acid to formulate the HFol diet, providing a total of 20 mg folic acid/kg diet. The normal dietary content of sucrose in the RFol diet is 100 g/kg diet [33], but because the folate mix used sucrose as a carrier (1 g folic acid/1 g sucrose), we reduced the manually added sucrose to only 99.982 g in the HFol diet to adjust for the 18 mg of sucrose from the 18 mg folic acid. The two diets were of the same energy density (3760 kcal/kg). Thus, except for the folic acid content, the rats were fed equivalent diets. Treatment diets and distilled water were provided *ad libitum*.

After delivery, all dams received the RFol diet and the litters were culled to 10 pups per dam. Littermate effect was controlled by using the dams (*n* = 10 per maternal dietary group) as the experimental unit as opposed to using a smaller number of dams, which would include several siblings from the same litter. This is important because rats from the same litter are more likely to follow the same developmental trajectory to each other than those from another litter [34], and often not controlled in studies that are similar in design to this one [35]. At 21 days of age, the offspring from both groups were sexed. Two female pups from each litter were weaned, and each pup was assigned to either the RFol or the HFol diet, producing four experimental groups: RFol-RFol, HFol-RFol, RFol-HFol, and HFol-HFol (*n* = 10 rat offspring per group, each from different litters). Only female offspring were studied due to two reasons. First, because this was the first study to examine the effects of gestational folate supplementation on bone development in early life, we wanted to be conservative and select the sex with the higher prevalence of osteoporosis. Second, male offspring were allocated to a separate study with different objectives, and bones could not be collected. 

Rats were housed individually in transparent cages, in a temperature-controlled environment (22°C ± 1°C) with a 12-hour dark-light cycle. Rats were weighed on a weekly basis starting from weaning until 17 weeks after weaning when female offspring were terminated by decapitation after an overnight fast. Food intake was measured three times per week from weaning until 17 weeks after weaning. At the end of study, due to being outliers one rat each was lost from 3 of the 4 groups (RFol-RFol, HFol-RFol, and RFol-HFol) during followup resulting in a final sample size of *n* = 9 offspring for those 3 groups, and *n* = 10 offspring for the fourth group (HFol-HFol). Femurs, intact lumbar vertebrae 1–3 (L1–3), and lumbar vertebra 4 (L4) were collected, separated, and cleaned of muscle and cartilaginous tissue before being stored at −80°C. The study was conducted in accordance with the guidelines established by the Canadian Council on Animal Care and approved by the Animal Ethics Committee at the University of Toronto, Canada.

### 2.2. Physical Dimensions

Bone physical dimensions were measured using an electronic precision vernier caliper (Cederlane Laboratories Ltd., Hornby, ON, Canada). The femur was measured for the following parameters: femoral length along the caudal-cranial axis, femoral width along the anterior-posterior axis (AP), femoral width along the medial-lateral axis (ML), femoral head and femoral neck along the superior-inferior axis, and distal epiphysis (knee joint) along the ML axis. L4 was measured for the following parameters: height along the caudal-cranial axis, body width along the AP axis, and body width along the ML axis. 

### 2.3. Bone Area, Bone Mineral Content, and Bone Mineral Density

The left femur, intact L1-3, and L4 were scanned in air at room temperature using dual-energy X-ray absorptiometry (Orthometrix pDexa sabre, Host Software version 3.9.4; Scanner Software version 1.2.0) to determine projected bone area, BMC, and BMD using a scanning resolution of 0.2 mm × 0.2 mm and a speed of 10 mm/s, as previously described [36]. The femur and vertebrae were imaged in the frontal plane.

### 2.4. Biomechanical Strength Testing of Femur and Lumbar Vertebrae

Three-point bending of the left femur at the mid-shaft site and compression testing of L4 were performed to assess biomechanical strength properties on these bones using a materials testing system (Model 4442 Universal Testing System; Instron Corp., Canton, MA, USA) and specialized software program (Instron Series IX Automated Materials Tester, Version 8.15.00; Instron Corp) as previously described [36]. Prior to testing, left femur and L4 were hydrated in physiological saline (9 g NaCl/L) for four hours at room temperature. During mechanical testing, femurs were fractured at the mid-shaft site. To prepare L4 samples for compression testing, the superior and inferior articular processes were removed with scissors. Then the top and bottom of the vertebrae were made parallel using a file to allow L4 to sit flat on the stainless steel disc. No movement of L4 was detected by visual inspection during testing.

### 2.5. Statistical Analysis

Data are presented as the mean ± SEM, and statistical analyses were performed using the SAS System (Version 9.2, SAS Institute, Cary, NC). Statistical significance was declared at *P* < 0.05. Body weight data were analyzed by PROC MIXED repeated measures ANOVA (dam diet, pup diet, and time as main factors). All other measures were analyzed by general linear model two-way ANOVA (dam diet and pup diet as main factors), followed by Tukey's pairwise multiple comparisons test to determine differences among groups. Outliers were identified by the use of Grubbs' test [37].

To correct for the effect of body weight as a confounder of the effect of folic acid on bone, analysis of covariance (ANCOVA) was applied to adjust for final body weight [38], followed by Tukey's pairwise multiple comparisons test. Similarly, femoral outcomes that have been shown to be associated with body growth (femoral BMC, femoral bone area, femoral peak load, and femoral stiffness) were adjusted for both body weight and femoral length [39].

## 3. Results

### 3.1. Body Weight and Food Intake

**Figure 1:**
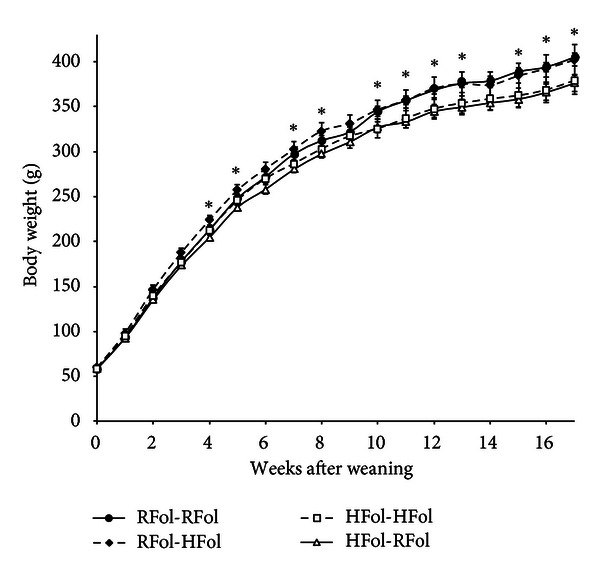
Weekly body weights of female offspring from weaning to 17 weeks after weaning. Data are presented as means ± SEM, *n* = 9-10 per group. Data analyzed by PROC MIXED procedure. Dam diet: *P* = 0.03; pup diet: NS; time: *P* < 0.0001; all interaction terms: NS *P < 0.05, HFol dam diet versus RFol dam diet.

There were independent effects of maternal diet (*P* = 0.03) and time (*P* < 0.0001) on body weights of female offspring, but not on food intake (data not shown). Those born to HFol dams had lower body weight than those born to RFol dams, starting from 4 weeks after weaning (Figure 1). At study termination, females born to HFol dams had 6% lower body weight (*P* = 0.03) compared to those born to RFol dams (377.9 ± 9.3 g versus 403.7 ± 8.6 g, resp.). Neither pup diet, nor interactions between dam, pup diets or time affected body weight (Figure 1) or food intake (data not shown). 

### 3.2. Femur Outcomes: Morphology, Bone Area, BMC, BMD; and Biomechanical Strength Properties

Folic acid content of the dam diets affected femur morphology, bone mineral content, and biomechanical strength, but the content of the pup diets and the interactions between the two diets had no effect on femur morphology (Table 2). When unadjusted for body weight, female offspring from HFol dams had smaller knee joint (*P* = 0.01) and smaller femoral length (*P* = 0.02), head (*P* = 0.03), neck (*P* = 0.04), and bone weight (*P* = 0.04) compared to those from RFol dams. AP and ML widths of femurs were not affected. However, after adjustment for body weight femur morphological measurements were no longer significantly different. Adjusted femoral length (*P* = 0.08) and knee joint (*P* = 0.07) approached statistical significance. The HFol pup diet had no significant effects on either unadjusted or adjusted femur morphology (Table 2). 

 Unadjusted femoral area (projected femoral area in the frontal plane) was affected by the folic acid content of the dam diet, but not the pup diet, such that the offspring of HFol dams had smaller projected area (*P* = 0.02) than those from RFol dams (Table 2). After adjustment, this difference was maintained (*P* = 0.03), but with a significant interaction (*P* = 0.01) between the folate content of the dam and pup diets. The interaction occurred because femoral area was lower in pups fed the HFol diet if they were born to mothers on the RFol diet, but tended to increase if the dams were fed the HFol diet. Femur BMC and BMD did not differ among groups (Table 2). 

 Neither maternal nor pup diets independently affected unadjusted femur peak load and stiffness (Table 2), or toughness, yield load, and resilience (data not shown). However, there were significant dam diet × pup diet interaction effects on femur peak load with adjustments and stiffness, with or without adjustments. The HFol diets resulted in lower adjusted peak load (*P* = 0.01) and lower stiffness (unadjusted, *P* = 0.02; adjusted, *P* = 0.002) (Table 2). The interaction occurred because peak load and stiffness tended to increase or be sustained in pups fed the HFol diet if born to HFol dams, but decreased if they were from RFol dams.

### 3.3. Lumbar Vertebrae Outcomes: Bone Area, BMC, BMD, Morphology, and Biomechanical Strength Properties

There were no significant effects of diets on unadjusted bone area, BMC, and BMD of intact L1-3 (data not shown), but projected L4 area approached significance (*P* = 0.06). However, offspring from HFol dams had lower L4 BMC (*P* = 0.02) and BMD (*P* = 0.04) than those born to RFol dams (Table 3), and these effects remained significant after adjustment for body weight (7%, *P* = 0.01 and 4%, *P* = 0.02, resp.).

Folic acid content of maternal diets had no effect on unadjusted morphology of L4 except for bone height (Table 3). Females from HFol dams had shorter L4 bone height (*P* = 0.04) than those from RFol dams, but this difference was no longer significant after adjustment. There were significant effects of pup diet on unadjusted L4 AP width (*P* = 0.02), but not height, ML width, or bone weight. Folic acid content of the pup diet was also a factor in development of L4 width. Those fed the HFol pup diet had smaller unadjusted L4 AP width (*P* = 0.02) than those fed the RFol pup diet, and this was maintained after adjustment (4%, *P* = 0.02). The folic acid content of both dam and pup diets had no effect on L4 peak load.

## 4. Discussion

The data from this study showed that a 10-fold increase in folate content of the diet during pregnancy reduced body weight gain of the offspring and both HFol maternal and pup diets affected formation and strength of some bones through independent and interactive effects. However, the results provided little evidence to support the PAR hypothesis, which proposes that matching pup with dam diets alleviates the adverse effects of maternal diets [32].

In contrast, increased folic acid in the diet of the dams and pups affected many characteristics of the femurs and lumbar vertebrae when the unadjusted data were analyzed. However, epidemiological [40, 41] and animal studies [38] have reported a relationship between body weight and bone health, indicating that body weight needs to be considered in assessing treatment effects on bone. After adjustment of dependent measures as appropriate for body weight [38] or body weight and femoral length [39], fewer parameters of bone were affected by folic acid additions. These findings were consistent with other studies that adjusted for body weight; maternal folate intake was positively associated with spinal BMD in children at 6 years [23] and 9 years [24] of age. Nevertheless, the adjusted results supported the hypothesis that maternal folic acid may affect bone health in offspring. 

 HFol maternal diets led to smaller projected femoral area in the offspring. After adjustments, interactions were found between folate content of the dam and pup diets. The combination of HFol dam and pup diets resulted in smaller femur bone area and lower peak load strength and stiffness of femur. The HFol pup diet independently resulted in lower L4 AP width, BMC, and BMD. This suggested that excess folic acid supplementation during pregnancy and early life may be detrimental to offspring bone health but contrasts with studies showing that maternal folic acid supplementation is beneficial to bone health during childhood [23–25] or aging [8–11]. However, some of the aforementioned studies did not measure the folate intake levels of the children (their postnatal diet) and therefore cannot exclude the possibility that the child's current diet may have had a role in modifying offspring bone health, as opposed to the influence of only the mother's diet during pregnancy [23]. 

We expected that changes induced by the HFol dam diet on body weight and bone health would be prevented if the offspring were weaned to the HFol pup diet based on the PAR [32]. The high folate content of maternal diets resulted in lower body weights of female offspring, as was previously shown in those born to dams fed only a 2.5-fold increase in folate [16]. It was unexpected that the differences in body weight were not explained by differences in food intake or caloric content: this suggests that other factors may have been involved, such as altered energy expenditure. However, this difference in body weight was not corrected by matching the folic acid content of the pup diet to the dam diet. This was in contrast with our recent study in which the obesogenic phenotype of offspring born to dams fed a high multivitamin maternal diet (10-fold the recommended vitamin levels) was prevented by feeding the offspring the same high multivitamin diet, or a diet high in folic acid alone (the HFol diet) [14]. Furthermore, matching the HFol pup diet to the HFol dam diet failed to attenuate the majority of effects of the HFol dam diet on bone health parameters. Only the lower femoral peak load and stiffness were attenuated and prevented, respectively, in the HFol-HFol group compared to the RFol-HFol group. It was not clear why the HFol pup diet protected femur biomechanical strength against the effects induced by the HFol dam diet, but did not protect against other effects. 

A limitation of the study was its short duration, because the focus was to examine the effects of HFol exposure *in utero* and in early life on bone health prior to attaining peak bone mass and therefore left uncertain their consequences to long-term bone health. To examine the effects of HFol diets on osteoporosis as a direct endpoint, future studies need to maintain rats to older ages. In addition, many comparisons were performed in assessing treatment effects, suggesting that some of these effects may have been declared significant due to chance alone. However, the multiway ANOVA combined with Tukey's mean comparison tests addressed the limitations of multiple comparisons and provided appropriate adjustments. Also, while larger sample sizes have been proposed as needed for bone studies, a strength of our study was that we controlled for potential littermate effect (i.e., animals from the same litter develop similar characteristics and health outcomes [34]), by selecting one female offspring from each dam (*n* = 9-10 offspring per dietary group, each offspring from different litters), as opposed to using several animals from the same litter. Finally, no mechanisms were examined. An interaction between metabolic responses to inadequate intake and bone metabolism has been described for folate deficiency, but not for excess intakes. Folate inadequacy results in increased homocysteine in blood and tissues, which stimulates osteoclast activity [42]. However, the effects of excess folate in the maternal diets may have been due to the role of folate in methylation and in mediating epigenetic changes in DNA and gene expression [15]. This has been shown in many tissues during development, including bone [43]. 

The results of this study in rats may be relevant to humans. Maternal intakes of 2.5-fold and 10-fold higher than requirement occur in women of child-bearing age or during pregnancy [27, 44, 45]. The basal dietary requirement for rats has been generally accepted to be 2 mg of folic acid/kg diet [33], and the equivalent for women is a recommended daily allowance (RDA) of 400 *μ*g/day of folate equivalents. The dose of 10-fold folic acid in this study was equivalent to an adult intake of 2000 *μ*g/day. Furthermore, studies have reported that pregnant women have folic acid intakes greater than the tolerable upper limit of 1000 *μ*g/day. More than 10% of pregnant women in a North Carolina population had a mean intake level above the tolerable upper limit [45], and pregnant women in Boston had a mean folic acid intake of 2.6-fold higher than the RDA (~1,050 *μ*g/day), of which women in the upper third quartile had a mean intake that was 3.4-fold higher than the RDA [44]. These high intake levels may be explained by new recommendations proposed for pregnant women to take folic acid supplements 2.75-fold (1,100 *μ*g/day) and 12.5-fold (5,000 *μ*g/day) higher than the RDA in order to protect against neural tube defects, and to protect women at high risk for folate deficiency [26–28]. 

## 5. Conclusion

In conclusion, folate in excess at 10-fold requirements fed to rat dams and their offspring affected area and strength of femurs, and BMD and BMC, but not strength of lumbar vertebrae in the offspring despite differences in body weight. However, after adjusting for body weight and femoral length, femoral midshaft strength was still compromised by the combination of high maternal folate intake and postweaning intake of folate at recommended levels. Furthermore, matching the high folate content of the maternal and pup diets protected femoral strength from this effect. These findings are of concern because of the increasing intakes of folic acid above requirements. Investigations into the long-term effects of folic acid supplementation during pregnancy on body weight and bone health of the offspring are required to provide insight into fetal and early life programming of health outcomes in the offspring.

## Figures and Tables

**Table 1:** Composition of diets.

Component (g/kg powdered diet)	RFol diet (AIN-93G)	HFol diet (modified AIN-93G)
Cornstarch^1^	529.5	529.5
Casein (>85% protein)^2^	200.0	200.0
Sucrose (added)^3^	100.0*	99.982*
Sucrose (from mineral mix)	21.4	21.4
Sucrose (from vitamin mix)	9.75	9.75
Sucrose (from folate mix)	0.0*	0.018*
Fat^4†^	70.0	70.0
Fiber (cellulose)	50.0	50.0
Mineral mix	13.6	13.6
Vitamin mix	0.25	0.25
Folic acid (from vitamin mix)	0.002	0.002
Folic acid (from folate mix)	0.0*	0.018*
L-cystine	3.0	3.0
Choline bitartrate	2.5	2.5
Tert-butylhydroquinone	0.014	0.014
Total Kcal/Kg	**3760**	**3760**

^1^Dyets, Inc. (Bethlehem, PA); ^2^Harlan Teklad (Madison, WI); ^3^Allied Food Service (Toronto, ON); ^4^Loblaws (Toronto, ON); ^†^Fat in the RFol and HFol diets was derived from soybean oil.

*Denotes differences between diets.

**Table 2:** Measurements of femur morphology, bone mineral, and biomechanical strength properties from female offspring at 17 weeks after weaning^1^.

Variables	Treatment groups				*P* value		
	RFol-RFol	HFol-RFol	RFol-HFol	HFol-HFol	Dam diet	Pup diet	Dam diet × pup diet
Whole femur							
Length (mm)							
Unadjusted	36.14 ± 0.17^ab^	35.88 ± 0.12^ab^	36.51 ± 0.21^a^	35.53 ± 0.35^b^	0.02	NS	NS
Adjusted*	36.03 ± 0.23	35.99 ± 0.20	36.43 ± 0.23	35.62 ± 0.21	0.08	NS	NS
AP width (mm)							
Unadjusted	3.20 ± 0.04	3.08 ± 0.05	3.09 ± 0.05	3.11 ± 0.09	NS	NS	NS
Adjusted*	3.18 ± 0.06	3.11 ± 0.06	3.07 ± 0.06	3.13 ± 0.06	NS	NS	NS
ML width (mm)							
Unadjusted	4.27 ± 0.05	4.10 ± 0.09	4.09 ± 0.09	4.04 ± 0.08	NS	NS	NS
Adjusted*	4.28 ± 0.08	4.11 ± 0.08	4.09 ± 0.08	4.04 ± 0.07	NS	NS	NS
Femoral head (mm)							
Unadjusted	4.08 ± 0.03	3.96 ± 0.04	4.07 ± 0.03	3.97 ± 0.07	0.03	NS	NS
Adjusted*	4.06 ± 0.05	3.98 ± 0.05	4.06 ± 0.05	3.99 ± 0.05	NS	NS	NS
Femoral neck (mm)							
Unadjusted	2.05 ± 0.04	1.97 ± 0.04	2.04 ± 0.03	1.93 ± 0.06	0.04	NS	NS
Adjusted*	2.03 ± 0.04	1.99 ± 0.04	2.02 ± 0.04	1.94 ± 0.04	NS	NS	NS
Knee joint (mm)							
Unadjusted	6.69 ± 0.04	6.54 ± 0.06	6.66 ± 0.04	6.53 ± 0.07	0.01	NS	NS
Adjusted*	6.67 ± 0.05	6.56 ± 0.05	6.64 ± 0.05	6.54 ± 0.05	0.07	NS	NS
Weight (g)							
Unadjusted	1.046 ± 0.026^a^	1.000 ± 0.031^ab^	1.038 ± 0.024^ab^	0.953 ± 0.037^b^	0.04	NS	NS
Adjusted*	1.035 ± 0.029	1.011 ± 0.030	1.029 ± 0.030	0.961 ± 0.028	NS	NS	NS
Bone area (cm^2^)							
Unadjusted	2.045 ± 0.010^a^	1.958 ± 0.045^ab^	1.993 ± 0.021^ab^	1.882 ± 0.041^b^	0.002	0.06	NS
Adjusted**	2.025 ± 0.018^a^	1.933 ± 0.018^b^	1.944 ± 0.018^b^	1.950 ± 0.017^b^	0.03	0.08	0.01
BMC (g)							
Unadjusted	0.487 ± 0.007	0.460 ± 0.008	0.463 ± 0.009	0.435 ± 0.017	0.08	NS	NS
Adjusted**	0.473 ± 0.010	0.453 ± 0.010	0.445 ± 0.010	0.460 ± 0.010	NS	NS	0.09
BMD (g/cm^2^)							
Unadjusted	0.236 ± 0.004	0.229 ± 0.002	0.232 ± 0.003	0.230 ± 0.005	NS	NS	NS
Adjusted*	0.235 ± 0.004	0.233 ± 0.004	0.231 ± 0.004	0.233 ± 0.004	NS	NS	NS

Femur midpoint							
Peak load (N)							
Unadjusted	145.06 ± 3.62	132.63 ± 2.79	135.67 ± 3.23	137.15 ± 5.96	NS	NS	0.10
Adjusted**	142.53 ± 3.48	134.73 ± 3.68	130.42 ± 3.27	142.19 ± 3.45	NS	NS	0.01
Stiffness (N/mm)							
Unadjusted	396.92 ± 14.88	354.50 ± 6.92	357.57 ± 5.85	372.23 ± 14.67	NS	NS	0.02
Adjusted**	390.43 ± 10.46^a^	359.88 ± 10.42^ab^	344.29 ± 10.31^b^	385.03 ± 9.79^a^	NS	NS	0.002

^1^Data are means ± (SEM); *N* = 9-10/group. Data were analyzed by 2-way ANOVA (dam diet and pup diet as main factors) followed by post hoc Tukey's test. Means with different superscripts in a row are significantly different, *P* < 0.05.

*Adjusted for body weight.

**Adjusted for body weight and femoral length.

**Table 3:** Measurements of lumbar vertebra 4 morphology, bone mineral, and biomechanical strength properties from female offspring at 17 weeks after weaning^1^.

Variables	Treatment groups				*P* value		
	RFol-RFol	HFol-RFol	RFol-HFol	HFol-HFol	Dam diet	Pup diet	Dam diet × pup diet
Height (mm)							
Unadjusted	8.34 ± 0.09	8.10 ± 0.10	8.24 ± 0.13	8.00 ± 0.12	0.04	NS	NS
Adjusted	8.29 ± 0.11	8.14 ± 0.11	8.20 ± 0.11	8.03 ± 0.10	NS	NS	NS
Body AP width (mm)							
Unadjusted	3.22 ± 0.03	3.20 ± 0.07	3.13 ± 0.04	3.06 ± 0.04	NS	0.02	NS
Adjusted	3.21 ± 0.05	3.21 ± 0.05	3.12 ± 0.05	3.07 ± 0.04	NS	0.02	NS
Body ML width (mm)							
Unadjusted	4.77 ± 0.05	4.78 ± 0.11	4.76 ± 0.07	4.60 ± 0.09	NS	NS	NS
Adjusted	4.73 ± 0.08	4.82 ± 0.08	4.72 ± 0.08	4.63 ± 0.07	NS	NS	NS
Weight (g)							
Unadjusted	0.378 ± 0.010	0.363 ± 0.009	0.367 ± 0.007	0.344 ± 0.016	NS	NS	NS
Adjusted	0.372 ± 0.011	0.369 ± 0.011	0.363 ± 0.011	0.348 ± 0.010	NS	NS	NS
Area (cm^2^)							
Unadjusted	0.709 ± 0.013	0.675 ± 0.008	0.680 ± 0.011	0.654 ± 0.023	0.06	NS	NS
Adjusted	0.703 ± 0.015	0.681 ± 0.015	0.676 ± 0.015	0.658 ± 0.015	NS	0.09	NS
BMC (g)							
Unadjusted	0.144 ± 0.004	0.137 ± 0.002	0.132 ± 0.003	0.127 ± 0.007	NS	0.02	NS
Adjusted	0.141 ± 0.004	0.139 ± 0.004	0.131 ± 0.004	0.129 ± 0.004	NS	0.01	NS
BMD (g/cm^2^)							
Unadjusted	0.203 ± 0.004	0.202 ± 0.002	0.195 ± 0.003	0.194 ± 0.005	NS	0.04	NS
Adjusted	0.201 ± 0.004	0.205 ± 0.004	0.193 ± 0.004	0.195 ± 0.003	NS	0.02	NS
Peak load (N)							
Unadjusted	415.09 ± 17.80	400.37 ± 14.73	379.36 ± 23.10	363.17 ± 26.63	NS	0.10	NS
Adjusted	406.50 ± 21.06	408.62 ± 21.03	372.57 ± 20.92	369.58 ± 19.84	NS	0.08	NS

^1^Data are means ± (SEM); *N* = 9-10/group. Data were analyzed by 2-way ANOVA (dam diet and pup diet as main factors) followed by post hoc Tukey's test. Data were further adjusted for body weight. Means with different superscripts in a row are significantly different, *P* < 0.05.

## Abbreviations

AP: Anterior-posterior
BMC: Bone mineral content
BMD: Bone mineral density
HFol: High folic acid (10-fold higher than recommended amount)
L1–3: Lumbar vertebrae 1–3
L4: Lumbar vertebra 4
ML: Medial-lateral
PAR: Predictive Adaptive Response hypothesis
RDA: Recommended daily allowance
RFol: Recommended folic acid amount.
